# Correction: ClC-2 knockdown prevents cerebrovascular remodeling via inhibition of the Wnt/β-catenin signaling pathway

**DOI:** 10.1186/s11658-023-00527-9

**Published:** 2024-01-03

**Authors:** Jingjing Lu, Feng Xu, Yingna Zhang, Hong Lu, Jiewen Zhang

**Affiliations:** 1 Department of Neurology, Henan People’s Hospital, No. 7 Wai-5 Road, Zhengzhou, 450052 Henan China; 2 Department of Urology, First Affiliated Hospital, Zhengzhou University, Zhengzhou, China; 3 Institute of Medical and Pharmaceutical Sciences, Zhengzhou University, Zhengzhou, China; 4 Department of Neurology, First Affiliated Hospital, Zhengzhou University, Zhengzhou, 450052 Henan China

**Correction** **:** **Cellular & Molecular Biology Letters (2018) 23:29** **https://doi.org/10.1186/s11658-018-0095-z**

Following publication of the original article [1], the authors informed us that there is in Fig. 3C. The pictures used in the AngII and AngII + Negative groups in Fig. 3C were repeated. Neither of these changes affects the results and conclusions of this study.

The correct Fig. 3 is given below:

**Fig. 3:**
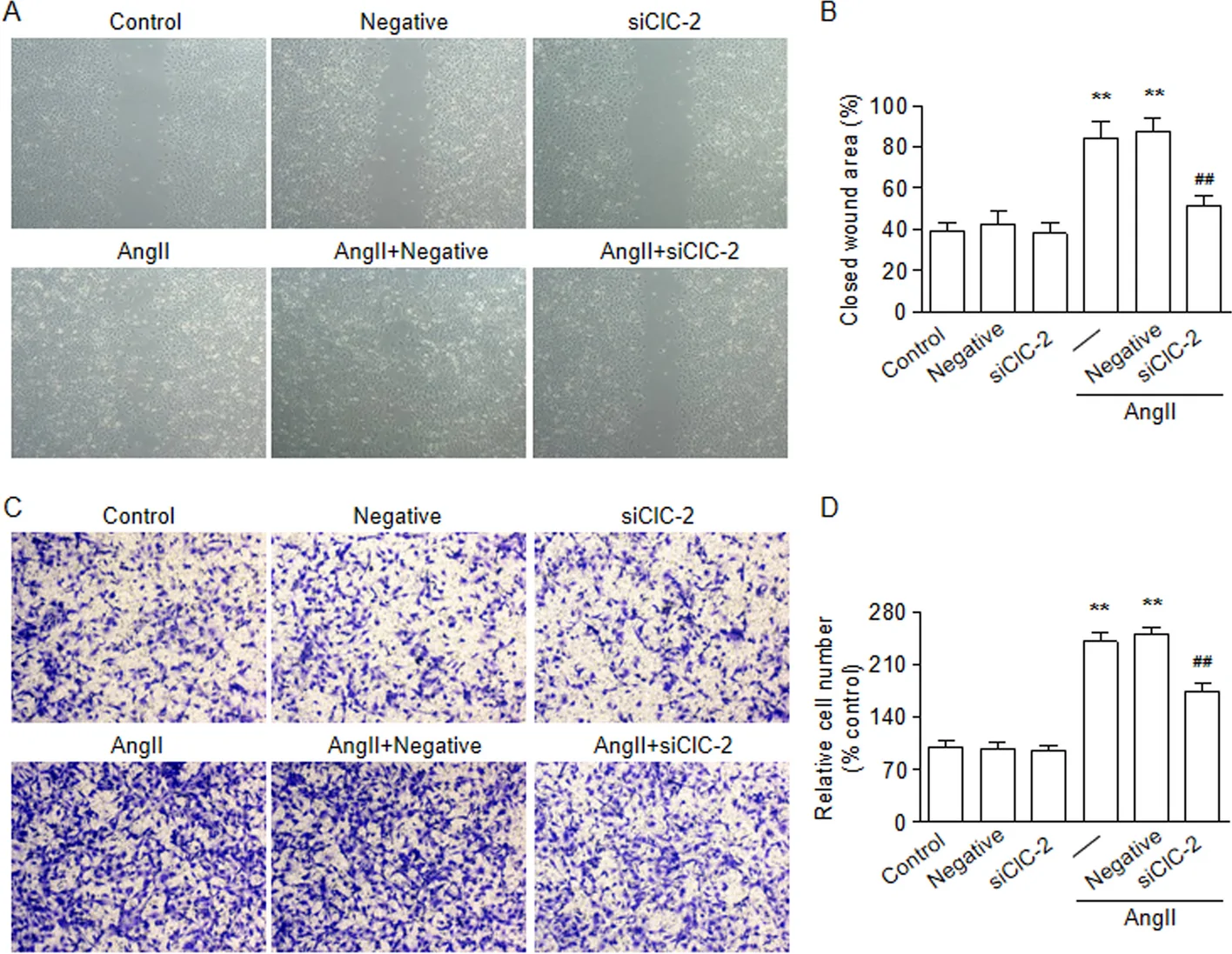
ClC-2 downregulation prevented AngII-induced HBVSMC migration and invasion. **a** HBVSMCs transfected with ClC-2 siRNA (siClC-2; 20 nM) or negative siRNA (negative; 20 nM) were subjected to angiotensin II (AngII) treatment (10 − 7 M). The wound healing assay was performed. Representative images are shown (× 100). **b** The quantification results for the wound closure. c HBVSMC migration was examined via transwell analysis. Representative images are shown (× 100). **d** The columns represent the relative numbers of invasive cells. **p < 0.01 vs. control, ^##^*p* < 0.01 vs. AngII alone, *n* = 6
